# Clinical Judgement, Treatment Decisions and Frailty Management in Older Cancer Patients: A Qualitative Study Exploring the Experiences of Radiation Therapy Staff

**DOI:** 10.1002/jmrs.70017

**Published:** 2025-08-16

**Authors:** Sofia Axelsson, Per Fessé, Per Fransson, Agneta Schröder, Antonis Valachis, Emma Ohlsson‐Nevo

**Affiliations:** ^1^ Department of Oncology, Faculty of Medicine and Health Örebro University Örebro Sweden; ^2^ University Health Research Centre, Faculty of Medicine and Health Örebro University Örebro Sweden; ^3^ Center for Research & Development Uppsala University/Region Gävleborg Gävle Sweden; ^4^ Sahlgrenska Academy, Institute of Health and Care Science University of Gothenburg Göteborg Sweden; ^5^ Department of Nursing Umeå University Umeå Sweden; ^6^ Department of Nursing, Faculty of Health, Care and Nursing Norwegian University of Science and Technology (NTNU) Gjøvik Norway; ^7^ Faculty of Medicine and Health, Department of Surgery Örebro University Örebro Örebro Sweden

**Keywords:** decision‐making, experiences, frailty, geriatric assessment, radiation therapy staff

## Abstract

**Introduction:**

Treatment of older cancer patients can be complex due to frailty that comes with age, and the benefits of radiation therapy for frail older patients are unclear. Radiation therapy staff play a crucial role in identifying and monitoring frailty and tailoring treatment. Research on radiation therapy in frail older patients is limited, and frailty assessments are not widely used in routine care. Understanding staff experiences with clinical judgement and frailty assessment is important for effective treatment. This study explored the radiation therapy staff's experiences of clinical judgement, treatment decision‐making, and managing frail older cancer patients.

**Methods:**

The study has an inductive design in which 12 specialist oncology nurses and four clinical oncologists working with cancer patients at four radiation therapy units across four counties in Sweden were interviewed. In total, 16 participants were interviewed for the study. Data were collected in semi‐structured individual interviews and were analysed using reflexive thematic analysis.

**Results:**

Three themes were identified from the interview analysis: *Putting the patient first*, *Care with integrity and humanity* and *Receiving support in treatment decisions*, along with nine sub‐themes. None of the participants was using any structured instrument for assessing frailty.

**Conclusions:**

Radiation therapy staff face significant and complex challenges when treating frail older cancer patients, which can lead to feelings of doubt and powerlessness. Awareness and use of structured frailty assessment are limited. Integrating structured frailty assessment could address the complex challenges experienced by staff by improving decision‐making, communication, and patient outcomes, contributing to more ethical and person‐centred care.

## Graphical abstract

Radiation therapy staff face complex challenges when treating older cancer patients. Awareness and use of structured frailty assessments are limited. Integrating structured frailty assessment could address the complex challenges. Need for further research to explore frailty assessment tools in radiation therapy care.

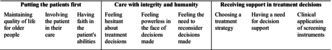

## 1 Introduction

Older people often experience illnesses or injuries associated with aging. The incidence rate of cancer increases with age, as does the incidence of frailty, with 25% of those aged 85 and over being frail [1, 2, 3]. Frailty is defined by the World Health Organisation (WHO) as ‘a clinically recognisable state in which the ability of older people to cope with every day or acute stressors is compromised by an increased vulnerability brought by age‐associated declines in physiological reserve and function across multiple organ systems’ (p. 8) [4]. Signs of frailty in older cancer patients include decreased physical activity, weakness, poor balance, and poor coordination, and lead to reports of higher symptom burden, reduced functioning, and lower quality of life (QoL) throughout the cancer trajectory [5]. These patients may have underlying morbidity and health‐related conditions that contribute to their frailty, increasing their vulnerability to adverse health outcomes such as increased mortality and reduced tolerance to cancer treatment, which makes treatment decisions in the care of older cancer patients challenging [6, 7, 8, 9, 10, 11].

The International Society of Geriatric Oncology (SIOG), European Association of Urology (EAU) and Royal Australian and New Zealand College of Radiologists (RANZCR) recommend making a structured, comprehensive geriatric assessment (CGA) including a mandatory assessment of frailty. This facilitates treatment decisions and allows treatments to be tailored to the patient, based on their degree of frailty [9, 12, 13, 14]. A structured CGA considers various parameters, such as comorbidity, polypharmacy, mobility and self‐perceived health status, and uses several instruments in the assessment to evaluate a patient's ability to cope with cancer treatment. As a comprehensive assessment is time‐consuming, a short geriatric assessment (GA) consisting of a short screening instrument can be used to screen for frailty and provide support in treatment decisions in the clinical setting [6, 7, 12, 15]. Health care professionals play a crucial role in caring for older cancer patients, from identifying frailty and making individualised cancer treatment decisions based on the patient's degree of frailty to monitoring frailty throughout their treatment, as frailty levels may change significantly during treatment [5, 16].

Radiation therapy (RT) is one of the most common treatment strategies for older cancer patients; although it remains unclear whether frail older patients benefit from RT, the treatment can cause side effects that can negatively affect QoL long term. Despite the importance of identifying frailty [17] the awareness and knowledge of structured assessment of frailty among RT staff can be limited [18]. Research on RT in frail patients and their need for personalised treatment options is lacking [8]. Frailty assessments, such as the CGA or GA, are not widely implemented in routine RT care globally.

To our knowledge, no previous studies have explored the experiences of RT staff in Sweden treating and managing frail older cancer patients in RT. Radiation therapy units in Sweden are staffed by clinical oncologists, specialist nurses in oncology and RT, nurses, and radiology nurses. The clinical oncologists prescribe the RT, while specialist oncology nurses, nurses, and radiology nurses manage the planning and delivery of RT and patient care before, during, and after RT. As a nurse in an RT unit in Sweden, you need either a master's degree in oncology care with a focus on RT, which gives you the title specialist nurse, or to have completed one course in oncology and one in RT lasting 30 weeks in total. Radiology nurses must take the same two courses, one for oncology and one for RT, as the nurses [19]. Before starting to work with RT, nurses in Sweden have typically gained experience working in other units, including oncology, surgery and infectious diseases departments. To successfully implement a structured frailty assessment in RT, it is important to understand the experiences of the care staff delivering the RT. Therefore, this study aimed to explore radiation therapy staff´s experiences of clinical judgement, treatment decision making, and managing frail older cancer patients.

## 2 Methods

### 2.1 Design

This study had an inductive qualitative design and used reflexive thematic analysis according to Braun and Clarke to explore and interpret patterns [20].

### 2.2 Recruitment

Purposive sampling was used to ensure variation in participants' gender, age, and time in the profession. Inclusion criteria were specialist nurses in oncology and clinical oncologists working with cancer patients at RT units across four counties in Sweden. Recruitment was conducted by authors SA and PF and two research nurses at altogether four RT clinics.

### 2.3 Participants

Seventeen individuals were invited to participate in the study: 13 specialist oncology nurses and four clinical oncologists. One specialist nurse declined participation for unknown reasons. In total, 16 participants were interviewed for the study.

### 2.4 Data Collection

Data were collected using semi‐structured individual interviews. To ensure consistency across topics, the participants were given a brief explanation of frailty and GA at the start of the interviews, and an interview guide (Table 1) was used with the initial question: *Tell me about your experience of a structured GA of older patients with cancer*. In addition, several follow‐up questions were asked. Participants were encouraged, through probing questions, to expand on their experiences. The interviews were conducted between November 2022 and April 2023 in secluded rooms at the clinics. The first author (SA), a doctoral student, conducted eleven interviews. The second author (PF) interviewed the remaining five participants from the first author's workplace. This was to allow the first author's colleagues to speak freely. Interviews lasted between 17 and 50 min. All interviews were audio‐recorded and were transcribed verbatim by independent professional transcribers, resulting in 120 pages of written data.

**TABLE 1:** Example from the interview guide.

Initial question	Tell me about your experience of a structured GA of older patients with cancer
Example of follow‐up questions	What would it be like for you with a structured assessment? What are the difficulties associated with the decision?
Example of Probing question	Can you elaborate on that? Can you tell me more about it?

*Note:* These questions were originally developed in Swedish, and what is presented here is the closest translation into English.

### 2.5 Analysis

The analysis commenced after all interviews were transcribed using the six phases of reflexive thematic analysis [20]. In the first phase, to familiarise herself and become immersed with the data, the first author listened to the audio recordings and read and reread all transcripts, making notes on initial observations. In the second phase, initial codes addressing the research question were generated by the first author from each transcript using NVivo 14 software. After the initial codes were generated, the authors examined the codes in the third phase, and broader patterns of meaning, potential themes, were developed that reflected patterns in the data. In the fourth phase, themes were reviewed and further developed, and subthemes were identified. In the fifth phase, a detailed analysis of each theme was conducted, and themes and subthemes were named to reflect the content of the data. In the final step, the analytical narrative and extracted data were woven together and contextualised to provide a comprehensive picture of the data (Table 2). Steps three to five in the analysis were conducted by the authors SA, AS, and EON, and the final step was conducted by all authors. During the analysis process, the authors strived to maintain reflexivity by remaining aware of our preunderstanding through continuous discussions. Five out of the six authors are nurses, three of whom specialise in RT. One of the authors is an oncologist.

**TABLE 2:** Examples of codes, sub‐themes and themes.

Code	Sub‐theme	Theme
So, it's the only thing that you formalise a little bit more in the performance status. But then, as I said, you might still have internally. so… you take more things into account (Ö4)	Choosing a treatment strategy	Receiving support in treatment decisions
That you get guidance on whether the patient will benefit from the treatment or not. Whether you do good or not (E4)	Having a need for decision support	Receiving support in treatment decisions
That you can estimate your or assess, answer questions about what your problems are a little bit and so, that you can focus on the right things, in the little time you have. Yes, but kind of make everything more efficient and that the patient gets the most out of it, like doctor's visit or nurse's visit or something like that (E1)	Clinical application of screening instruments	Receiving support in treatment decisions

### 2.6 Ethics

The study has undergone an ethical review and has been approved by the Swedish Ethical Review Authority (ref No: 2021‐06939‐01). Prior to the interviews, the participants received both oral and written information about the study and voluntarily signed an informed consent form. In addition, participants were informed of their rights to withdraw from the study at any time. The Consolidated Criteria for Reporting Qualitative (COREQ) checklist was used to guide reporting the study [21].

## 3 Results

Three themes were identified from the interview analysis: *Putting the patient first*; *Care with integrity and humanity*; and *Receiving support in treatment decisions*, along with nine subthemes (Table 3). At the time of the interviews, none of the participants were using any structured instrument for assessing frailty.

**TABLE 3:** Theme and sub‐theme.

Putting the patients first			Care with integrity and humanity			Receiving support in treatment decisions		
Maintaining quality of life for older people	Involving the patient in their care	Having faith in the patient's abilities	Feeling hesitant about treatment decisions	Feeling powerless in the face of decisions made	Feeling the need to reconsider decisions made	Choosing a treatment strategy	Having a need for decision support	Clinical application of screening instruments

### 3.1 Putting the Patient First

#### 3.1.1 Maintaining Quality of Life for Older People

In addition to giving consideration to clinical judgement, assessment, cancer type and intent and fractionation, the participants felt that it was important to consider the older and frail patient's life outside the hospital, their wellbeing and QoL. It was important to ensure that the treatment would provide more benefit than harm. The participants expressed that the burden of treatment must be considered and that symptomatic treatment for a frail older cancer patient should be prioritised to maintain or improve QoL. Care where older frail patients received unnecessary or unsuitable treatment that led to a decline in their QoL was perceived as a failure.

The patient can receive treatment for symptom relief in the time left, giving greater QoL than a curative treatment [which is associated] with significant discomfort. (V2)

#### 3.1.2 Involving the Patient in Their Care

The participants described that it was important to involve the patient in their care through including them in discussion and ensuring that they were treated as a person. This approach aimed to provide the best care tailored to each patient's needs and foster a comfortable environment for open communication between the patient and the RT team.

Geriatric screening instruments were perceived as a potential support for improving communication with the patients and their significant others, helping to better examine the patients and understand their wishes, needs and preferences, and involving them more fully in their care.

It's important that the patient can take part and give their approval of the treatment. (Ö3)

#### 3.1.3 Having Faith in the Patient's Abilities

The participants believed that patients' willingness to undergo RT was influenced by how the patients perceived their own capacity and ability to tolerate and manage RT. The participants sensed that the patients' perception of frailty and wellbeing was influenced by their social network. The participants reported that they considered the patients' perception of their own ability in their assessment. They emphasised the importance of considering all factors that influence an older patient's self‐perception, emotions and sense of physical strength, while treating each patient as a person and incorporating this understanding into their clinical judgement and assessment. They commented that, to qualify for RT, older frail patients sometimes tried to appear stronger than they felt.

What the patient can tolerate and handle depends on their own perception. Some are willing to take the negative aspects; others are not. (Ö4)

### 3.2 Care With Integrity and Humanity

#### 3.2.1 Feeling Hesitant About Treatment Decisions

Participants described how they found the clinical assessment of older cancer patients prior to RT to be complex, leading to feelings of doubt and uncertainty regarding treatment decisions. This was particularly challenging when working with older patients receiving palliative care, patients with dementia, those treated for brain tumours or patients perceived as frail. Balancing treatment benefits, side effects and the impact on QoL created feelings of reluctance and uncertainty among the participants, who often struggled with the decision‐making process.

It went a lot faster than we thought it would and we haven't been able to put our finger on it, even though we've thought and tried … So it's … it can be difficult to estimate the time and to know how the disease will develop. (Ö1)

#### 3.2.2 Feeling Powerless in the Face of Decisions Made

The participants described feelings of powerlessness regarding treatment decisions. They perceived that RT was used to ‘do something’ for the patient and that older palliative patients were sometimes planned to receive long‐term RT and then did not live long enough to experience its benefits. The participants often experienced that older patients were rarely considered to be too ill, too tired, or too old for RT, which led to ethical considerations about an appropriate and patient‐centred treatment.

Instead of giving him maybe a treatment for symptom relief and following up on that […], so he could have kept going a little longer. But it wasn't long after the treatment that it ended. I think that is a failure regarding intention. (V2)

#### 3.2.3 Feeling the Need to Reconsider Decisions Made

Feelings of needing to reconsider and question treatment decisions, especially regarding palliative treatment for frail older cancer patients, were described by the specialist oncology nurses. They expressed that they used their clinical judgement to assess a patient's wellbeing before starting RT. All participants experienced that they could discuss treatment decisions with colleagues if they deemed the patient's condition too poor to start treatment.

The specialist oncology nurses experienced that clinical oncologists listened to their input and relied on their clinical experience and judgement before making their own clinical assessment of the patient's condition to determine whether the treatment was appropriate; existing treatment decisions often remained unchanged.

Because especially, as palliative patients … I think their condition is too poor and you question whether they will really get to benefit from treatment. (Ö3)

### 3.3 Receiving Support in Treatment Decisions

#### 3.3.1 Choosing a Treatment Strategy

The participants discussed their approach to treatment strategies for older cancer patients, noting the differences between curative and palliative care. They experienced that RT was often chosen for older patients regardless of factors such as frailty or overall health status. A gentler approach to curative treatment was usually preferred, with shorter courses and a palliative approach for symptom relief in frail patients. Decision‐making was experienced as more challenging with frail patients compared with patients in better health. The participants emphasised the importance of clinically assessing the patient's overall health and carefully weighing the benefits versus risks of treatment for frail patients. They described relying on the patient's medical record and performance status, as well as an examination of the patient, and their own clinical judgement and sense of the patient's ability to cope with treatment when choosing a treatment strategy. Collaboration between the clinical oncologist and specialist oncology nurses was essential in tailoring the treatment to a patient's needs, providing a more holistic view of the patient's capabilities. However, communication gaps between departments regarding palliative care strategies were identified as an area requiring improvement.

There might be more discussion for palliative care on benefits versus risks, while curative care might involve focusing more on cheering on the patient to get through this, that it will likely benefit them in the future. (G1)

#### 3.3.2 Having a Need for Decision Support

*Determining the best treatment for frail older patients could be challenging, according to the participants*. They reported that they often sought support for uncertain treatment decisions, especially in cases where the optimal strategy was unclear. They believed that decision support tools, such as frailty assessment instruments, could add valuable guidance in planning care tailored to the patient's needs during treatment to clarify treatment decisions and minimise feelings of uncertainty.

It confirms that you're on the right track, that this is my sense, and my assessment is this, and it also shows that. It becomes an added dimension and safety, it's patient safety. (E1)

#### 3.3.3 Clinical Application of Screening Instruments

Screening instruments for GA were seen as a possible aid in the assessment of older cancer patients prior to RT and in treatment decisions and could potentially provide assistance in understanding a patient's health status, determining their ability to tolerate curative treatment, or deciding whether a palliative approach would better maintain quality of life. They recognised that conducting a baseline assessment could be beneficial in monitoring health over time, allowing for preventative treatment and minimising side effects that could negatively affect the patient. In addition, the use of a frailty assessment instrument in a structured way was seen as beneficial in providing a consistent assessment of a patient's health and suitability for RT, regardless of who carried out the assessment. However, participants expressed the need for knowledge and training in the use of a frailty assessment instrument if such instruments were to be implemented in clinical RT practice.

So I think it would be good to have more structured assessments. Because when you don't have that, it's more fluid, it's more subjective; yes, I think it would be more fair to use the same one. (Ö2)

The participants reflected that a frailty assessment instrument could provide structure, especially for complex palliative treatments with unclear guidelines. Frailty assessments could possibly enhance communication between colleagues regarding treatment decisions. They might facilitate communication with older cancer patients and their significant others, enabling patients to make the most of their consultations with care personnel. However, concerns were raised about disadvantages, and scepticism was expressed about the clinical benefits of using such instrument. The participants identified potential disadvantages of relying on a frailty assessment instrument, including the risk of overreliance on the instrument, and not fully considering the patient's individual circumstances.

… that you estimate your, or assess, answers to questions regarding what you're having problems with a bit, so that you can focus on the right things, in that little time. Really, to make everything more efficient and so that the patient gets as much as possible out of it, for example a visit with a doctor or a nurse. (E1)

## 4 Discussion

This study aimed to explore RT staff's experiences of clinical judgement, treatment decision making, and managing frail older cancer patients. Participants were not in a position to directly answer the initial question, even though fragility and GA were explained before the interview. This may reflect the fact that none of the participants were using structured instruments to assess frailty at the time of the interviews. Instead, they focused on other factors that highlighted the need for GA, including general considerations regarding frailty in older cancer patients and the difficulties associated with treatment decision‐making. This can be attributed to the participants limited knowledge and awareness of GA and the absence of structured instruments to assess frailty in older cancer patients scheduled for RT in Sweden. The main findings of this study highlight the complexities RT staff face when assessing and treating frail older cancer patients. These findings reveal that staff often experience significant challenges in making a clinical judgement and treatment decision, and tailoring care strategies to maintain patients' QoL. The lack of implementation of structured instruments such as screening GA instruments contributes to feelings of uncertainty and ethical stress in decision‐making. The participants emphasised the need for person‐centred care and interprofessional collaboration.

The aim of this study aligns with previous research identifying challenges in decision‐making for older cancer patients with multimorbidity. For example, Seghers et al. reported similar difficulties in balancing treatment benefits and risks [22]. While earlier studies, such as by Hamaker et al. emphasise the utility of GA instruments [23], our study uniquely highlights the need for support in the decision‐making process using a GA instrument and identifies a lack of awareness and training in Swedish RT settings, indicating a gap in implementation readiness.

Our study also reports ethical stress among RT staff, resonating with findings from Morris et al. and Naseer et al. regarding insufficient preparation for geriatric oncology [18, 24]. Unlike Komatsu et al.'s study, which focuses on nurse‐led decision support [25], this study emphasises the need for interprofessional collaboration and tailored training to address these gaps effectively.

### 4.1 Strengths and Limitations

The qualitative design provided in‐depth insights into the experiences of RT staff, uncovering nuanced challenges and potential solutions. Diverse sampling from four RT clinics across Sweden ensured a range of perspectives from both nurses and clinical oncologists. Thematic analysis allowed for a comprehensive interpretation of the data, supported by rigorous methodological steps to enhance trustworthiness.

### 4.2 Trustworthiness

The study's trustworthiness was strengthened by Lincoln and Guba's four key aspects: credibility, transferability, dependability and confirmability [26]. This was achieved through a systematic analysis process. The first author's review and first‐hand interpretation of the interviews during the initial analysis while three authors, SA, AS, and EON, collaborated on coding and thematisation. Data extracts from multiple participants were incorporated into the Result section to support the themes, enhance nuances and thereby effectively communicate the findings, giving the reader a deeper understanding of the identified themes. Two of the authors had no prior experience with RT, which strengthened the reflexivity in the study.

To minimise subjectivity in data collection and analysis, bearing in mind the first author's dual role as researcher and RT nurse, all interviews were conducted by the first author except for those held at the RT clinic where she is employed.

The uneven gender distribution and predominance of specialist nurses in the sample may limit the generalisability of the findings to other professional groups. However, the majority of the workforce in RT in Sweden is female, and a significant proportion of RT staff are nurses. Participants' limited knowledge of structured GA instruments may have influenced the depth of discussions regarding the clinical utility of such screening instruments.

### 4.3 Clinical Implications and Future Research

The findings underscore the need for integrating GA into RT care to improve decision‐making, enhance communication and support patient‐centred outcomes. Tailoring treatment strategies based on frailty assessments can mitigate side effects, improve QoL and ensure ethical treatment decisions. Structured GA instruments can also serve as a unifying framework for interprofessional collaboration, for example by involving geriatricians in multidisciplinary conferences before treatment decisions are made for frail older cancer patients, thus promoting equitable care. The study further suggests that ethical stress experienced by RT staff stems from a lack of clear guidelines and tools, leading to a reliance on subjective judgement. This finding highlights the importance of fostering a supportive clinical environment where staff have access to training, decision support systems, and opportunities for collaborative deliberation to reduce uncertainties and enhance care quality.

Future research should explore the following: collaboration with geriatricians in the assessment of older cancer patients, and the development and implementation of a nurse‐led GA in RT settings with a focus on feasibility and impact on treatment outcomes; evaluating the effectiveness of training programmes aimed at enhancing RT staff's awareness, knowledge, and skills in frailty assessment; investigating the decision‐making processes in diverse cancer populations to identify tailored strategies for different clinical contexts and patients' needs; and examining the long‐term impact of integrating structured GA into RT care for older patients, particularly on QoL, treatment efficacy and patient‐centred outcomes.

## 5 Conclusions

Radiation therapy staff face significant, complex challenges in treating frail older cancer patients. These challenges are often compounded by limited awareness and implementation of structured GAs. Integrating GA into clinical practice has the potential to address these challenges by enhancing decision‐making processes, improving communication, and optimising patient outcomes. However, successful implementation requires targeted training, interprofessional collaboration and strong managerial support. This study highlights the urgent need for further research to explore the feasibility, usability and clinical outcomes of GA in RT care, ultimately contributing to more ethical and person‐centred oncology practices.

## Ethics Statement

The study was approved by the Swedish Ethical Review Authority (Ref. No: 2021–06939‐01).

## Conflicts of Interest

The authors declare no conflicts of interest.

## Data Availability

The data that support the findings of this study are available from the corresponding author upon reasonable request.
